# There’s no smoke without fire!

**DOI:** 10.1590/0102-311XEN103823

**Published:** 2023-10-09

**Authors:** Liana Oighenstein Anderson, Sonaira Silva, Antônio W. F. Melo

**Affiliations:** 1 Centro Nacional de Monitoramento e Alerta de Desastres Naturais, Ministério da Ciência, Tecnologia, Inovações e Comunicações, São José dos Campos, Brasil.; 2 Universidade Federal do Acre, Cruzeiro do Sul, Brasil.

Amazon deforestation data are used as a gauge, at the national and international levels, to indicate the current situation of the political management of the control of and combat against this process, which is usually widely disseminated in the media. Due to the weakening of environmental policies in recent years, there was a forecast that deforestation for the year 2020 ^1^ would be the highest of the decade, above that of 2019, which exceeded 10,800km^2^ ^1^, the highest rate since 2008. Although 2020 had a slightly lower rate than 2019, deforestation in 2021 and 2022 exceeded 12,000km^2^ ^2^, which again featured prominently in global media. Recently, the Yanomami crisis revealed another growing threat to Amazonian life: the push of mining activities in the region. Estimates point to increased mining rates mainly after 2010, and 2020 data showed that the total mining area exceeded the industrial mining area ^3^. The negative impacts - beyond social and cultural ruptures caused to indigenous peoples - include increased disease rates, environmental contamination, and food insecurity ^4^.

The advance of deforestation reveals a small part of the socio-environmental problem related to the Amazonian forests. A recent study ^5^ quantified that fire, forest fragmentation and logging between 2001 and 2018 have already impacted more than 5.5% of the forests in the entire Amazon basin, and this extension corresponds to 112% of the total area deforested in that period. If we add to this list of forest degradation vectors the occurrence of extreme droughts, the area increases to 38% of the remaining Amazonian forests.

It is widely known that fire is the main instrument for disposing of biomass after clear-cutting the forest, and that it causes a series of negative socioeconomic and environmental impacts. For example, on the global scale, greenhouse gas emissions from slash-and-burn practices and wildfires directly affect the rainfall and temperature regime and, on the regional scale, directly generate air pollution, thus affecting air quality ^6^ ^,^ ^7^. Locally, the negative impacts of fires include the degradation of soils and forests, the imposition of restrictions and losses on those who depend on them, affecting their properties, public infrastructure or even services ^8^. However, it is much less known that areas with forest fragmentation ^9^, logging ^10^, and forest areas that have already been affected by fire are more susceptible to new fires ^11^. Moreover, extreme droughts, which have intensified and become more recurrent due to climate change ^12^, amplify the extent and magnitude of fires ^7^. This means that even if deforestation rates are controlled, there are still all the other forcings that lead to the occurrence of fires present in human practices and Amazonian landscapes ^13^, and these have increased over this century ^14^.

The exposure of the population to smoke has several implications, both in the short and long terms, ranging from allergic-respiratory crises to cancer, impacting mainly children and the elderly. Smoke from slash-and-burn practices and forest fires is generally composed both of primary pollutants - such as particulate matter (PM), carbon monoxide (CO), nitrogen oxides (NOx), and non-methane organic compounds (NMOC) - and precursors of secondary pollutants, which include ozone (O_3_) and other secondary organic aerosols (SOA) ^15^. Several studies have demonstrated the relation between fine particulate matter (PM_2.5_) and increased hospital admissions due to respiratory problems in the Amazon region ^16^ ^,^ ^17^.

However, it should be noted that studies quantifying the impact of air pollution from slash-and-burn agriculture and fires face methodological challenges. The first refers to the detection of atmospheric aerosols, their dispersion in space, in the air column, and in time, as the duration of exposure of people in number of hours, days, weeks, etc. On the other hand, the search for these relations with cases of the number of people affected, usually attributed to the number of deaths and hospitalizations due to respiratory problems, are underestimated, either in the short-term time scale, due to the lack of registration of the number of people who seek self-medication to treat symptoms, or the problems of lack of health care units or difficult access. Records of outpatient care are limited, and in general only hospitalizations are counted, and in the long-term time scale, there is a bottleneck in studies that quantify cases in which recurrent exposure to pollutants can lead to the development of cancer, and that long-term exposure to smoke can reduce life expectancy by more than two years ^18^. It is estimated that, only between June and October 2019, the cost of hospitalizations in the Brazilian Unified National Health System (SUS) in the Amazon region associated with fires was approximately BRL 2.6 million ^19^, which excludes all other economic impacts, whether on the lives of the people who were hospitalized, which may compromise their income, but also on the entire production of goods and provision of services compromised by the illness of the population.

One of the main important aspects to be considered, both to measure and attribute responsibility for the impacts of fires on air quality, and to establish technical and political guidelines to foster a culture of risk perception as to exposure to them, refers to the availability of near real-time monitoring data. However, there is a lack of resources for the purchase and maintenance of sensors, as well as to establish and operate air quality monitoring networks throughout the country. A survey ^20^ published in 2021 showed that only ten states and the Federal District monitor air quality, with a network of 371 sensors, 80% of which are located in the Southeastern region of the country. This data gap leads to a situation of unpredictability as there are no quantitative bases so as to understand the magnitude of this growing problem. Technological advances in the development of low-cost sensors have been presented as an alternative, both for monitoring air quality and as a strategy for citizen science and social engagement ^21^.

An initiative that contributed to advances in the understanding of seasonality and human exposure to smoke was the network of low-cost sensors integrated with the Internet of Things (IoT) for on-site monitoring of particulate matter, started in the state of Acre and then expanded to Peru and Bolivia ^22^. Another innovative aspect of this monitoring network was the partnership established between researchers and the Public Prosecutor’s Office of the state of Acre, which enabled the expansion of the network to the other states of the Brazilian Amazon ^23^. This quantitative data collection network for the state of Acre showed that, between 2019 and 2022, only in the municipality of Brasiléia there was an annual average of 77 days with higher particulate matter concentration in the air than that established by the World Health Organization (WHO): 15μg/m^3^ in 24 hours. Other nine municipalities located in Acre also presented an annual average of more than two months of exposure to this type of pollution during this period ^24^. This critical diagnosis shows that 19 of the 22 municipalities in Acre had, on average, more than 30 days with values harmful to health. For the urban perimeter and surroundings of the city of Rio Branco, it was estimated that the adoption of a zero-hectare limit for slash-and-burn areas would result in avoiding 1,393 cases of hospitalizations due to acute respiratory diseases per year during the peak slash-and-burn months ^25^.

Pollution from slash-and-burn practices and forest fires is not an isolated factor. Since the first months of 2020, the COVID-19 pandemic has added another factor threatening the world and especially the Amazon region, where several social and environmental vulnerabilities are significant in relation to other regions of the country. For example, the mortality rate of indigenous people infected with the coronavirus was estimated to be about 150% higher than the Brazilian average, showing a greater vulnerability of this population ^26^. In January 2021, the collapse of the health care system in Manaus was staggering, with hospitals lacking oxygen and patients moved to other states, cemeteries with no space for new graves, and mass graves dug with backhoes, revealing unprecedented political and institutional vulnerability. Amid this situation, an external threat is added: in February 2021 a great flood begins in the west of the Amazon, flooding the state of Acre and reaching the central region of the Amazon, in Manaus, in June of the same year. This conjuncture is an example of how compound threats, such as COVID-19 and flooding, or COVID-19 and wildfires, can intensify the risks of each of these factors in isolation, thus amplifying the health risks in all cases.

However, in this recipe for socio-environmental construction of multi-threats to human health, we can still put more ingredients. Observational data show a rainfall reduction of 34% in the east and 20% in the west of the Amazon during the dry season months compared to 40 years ago. Temperature data show an increase for the same season and period ranging between 2.5ºC in the south of the biome, 1.7ºC in the west and 1.9ºC in the east ^27^. Combined to that, there is the advance of deforestation and increasing forest degradation toward the interior of the forest, exposing the population to new vectors of diseases such as mosquitoes and other types of viruses, in addition to jointly providing a more favorable environment for the occurrence of fires.

In 2023, we will face El Niño, which causes droughts and/or increased temperature in some regions of the Amazon, and data indicate that it should be of greater intensity than the historical average, developing over the coming months. We already know of the high rates of deforestation in recent years, indicating that these areas will burn, and could turn into large fires. We observe the challenges of the current government in keeping the promise of resuming the environmental agenda, still with limited success and established regressions. At the same time, the Amazon Summit assembly is planned for August this year in Belém (Pará State) and there is confirmation of the United Nations Climate Change Conferences (COP 30), which will also be held in Belém in 2025 - amid discussions on oil drilling at the Amazon river mouth with support from all the region’s governors. In this political seesaw, in which a fragmented vision predominates, separating environmental issues from economic and social issues, the Brazilian State continues without integrated short- and long-term plans to enable sustainable, socially and environmentally fair development for the Amazon and for Brazil with clear indicators and goals and guaranteed resources, subject to monitoring by society.
